# The Relaunch of *Microbiology Spectrum*

**DOI:** 10.1128/spectrum.00396-21

**Published:** 2021-06-09

**Authors:** Christina A. Cuomo

**Affiliations:** a Infectious Disease and Microbiome Program, Broad Institute of MIT and Harvard, Cambridge, Massachusetts, USA; Broad Institute

## EDITORIAL

The journal *Microbiology Spectrum*, published by the American Society for Microbiology, has relaunched as an open-access journal with a broad scope across microbiology research. Pivoting from publishing monographs such as review articles, *Spectrum* now welcomes all types of microbiology research studies that have been carried out in a rigorous way and advance knowledge in the area of focus, without consideration of novelty or impact. Central to the journal’s mission is promoting open science, reproducibility, and transparency in the review process.

*Microbiology Spectrum* has relaunched with a new scope of research across microbiology. While this includes the areas covered by many ASM journals, a notable distinction is that *Microbiology Spectrum* will not gate manuscripts based on the perceived impact or novelty of a study. Requiring such a threshold for publication can involve subjective assessments on the part of reviewers and editors, and this can limit the publication of high-quality work. Similar to other journals, *Microbiology Spectrum* will expect that manuscripts clearly describe the study’s rationale, design, methods, results, and interpretation, including limitations, with clear and concise writing.

The wide scope across microbiology and lack of impact criteria for publication expand the options for most authors within ASM publishing. In addition to being submitted directly to *Microbiology Spectrum*, manuscripts can be transferred from other ASM journals. For manuscripts transferred with reviews, editors seek to provide a decision based on the provided reviews and response of the authors, avoiding additional cycles of review when a paper is resubmitted. For other manuscripts, editors at *Microbiology Spectrum* will evaluate the paper and decide whether to solicit reviews.

*Microbiology Spectrum* will publish all types of reports that document rigorous work. These include replication studies that provide additional support for findings, as well as negative results that reject a study hypothesis. Studies could focus on a deeper investigation of previously published work, such as independent analyses of large data sets. In addition to research studies, methodological advances and protocols are also welcome.

*Microbiology Spectrum* supports these publication types and practices as part of a central ethos to bolster support for open science. Open and early data release is particularly urgent where the results may directly impact public health decisions, such as viral genome sequencing during the COVID-19 (coronavirus disease 2019) pandemic. More broadly, all research can be accelerated by the use of standard repositories, so that advances in one study can be easily built on or validated by other researchers. ASM has outlined an open data policy (https://journals.asm.org/open-data-policy) that authors at *Microbiology Spectrum* will need to follow to make it easy to find and reuse data and metadata. We also seek to promote papers that take additional steps to support the reproducibility of the results, such as providing walk-throughs or interactive notebooks in addition to making software or code available, such that users can easily take data from processing all the way to table or figure generation. For transparency in the review process, authors will have the option to publish reviews for their manuscript from *Microbiology Spectrum* along with the paper, which adds the context of issues raised and how they were addressed by the authors to the publication record. The wider publication of reviews may help ensure that reviewer feedback is carefully considered and considerate and will provide a wider view into the review process for early-career scientists. *Microbiology Spectrum* also welcomes manuscripts posted to preprint servers, and we are interested in considering how the review process can interface with preprint servers moving forward.

As part of our commitment to openness, we are building the editorial board at *Microbiology Spectrum* not only via direct invitations but also through open calls for those interested in supporting the mission of the journal. Open calls for editors are a needed change from reliance on personal networks and visibility in selecting editors. While a major factor in recruitment or selection is the expertise to cover needed areas of microbiology, an underlying principle is to build an editorial board that is diverse in terms of race and ethnicity, as well as geography, institution or employer, and career stage. The differences that people bring to the board are highly valued, such as different experiences with the review process and different ideas about priorities for the journal. For early-career stage scientists, we will soon open a call for those interested in serving as reviewers and encourage scientists at the postdoctoral level to apply. We look forward to working with other ASM programs to support building expertise as reviewers.

Moving forward, *Microbiology Spectrum* will seek to incorporate new practices that support strengthening the scientific record and promote open science. Many journals are experimenting with new publishing initiatives, and with our relaunch, we have a platform to pilot those that make review more transparent and equitable and improve the publication process for authors, reviewers, and readers. At its core, the mission of *Microbiology Spectrum* is to support microbial research and the microbiology community, and we embrace a culture of open discourse to chart a course forward.

